# Physicochemical Properties of Bread Partially Substituted with Unripe Green Banana (*Cavendish* spp.) Flour

**DOI:** 10.3390/molecules26072070

**Published:** 2021-04-03

**Authors:** Pakathip Thakaeng, Thitirat Boonloom, Saroat Rawdkuen

**Affiliations:** 1 Program in Food Science and Technology, School of Agro-Industry, Mae Fah Luang University, Chiang Rai 57100, Thailand; 5731401051@lamduan.mfu.ac.th (P.T.); 5731401017@lamduan.mfu.ac.th (T.B.); 2 Unit of Innovative Food Packaging and Biomaterials, Mae Fah Luang University, Chiang Rai 57100, Thailand

**Keywords:** unripe green banana, *Cavendish* spp., wheat flour substitution, bread, gluten-free, functional properties

## Abstract

This study aimed to utilize unripe green bananas obtained from those that were graded as unacceptable for export. Bread was selected as the product model for the application of banana flour. As carbohydrates and other functional active compounds make up the main composition of green bananas, unripe banana flour (UBF) was prepared and characterized. The chemical composition, physico-chemical properties, and functional properties of UBF, as well as its application in bread for wheat flour (WF) substitution at different levels, were investigated. Quality attributes of the bread were determined. High carbohydrate (89%), total dietary fiber (7%), ash (2%), potassium content and radical scavenging activity were found in UBF bread, while protein (15%) and fat contents (0.9%) were higher in WF bread (*p* < 0.05). Starch granules of different sizes and shapes (round, long and oblong) were observed in the starch from UBF bread. Solubility, swelling power, and the water absorption capacity of WF bread were greater than UBF bread (*p* < 0.05). The gelatinization enthalpy (ΔH) was 0.69 and 5.00 J/g for WF and UBF, respectively. The rapid viscoanalyzer (RVA) pasting profile showed that UBF bread had a higher pasting temperature, peak viscosity, breakdown, and final viscosity than WF bread (*p* < 0.05). Increasing the level of UBF caused an increase in bread hardness and a decrease in loaf volume (*p* < 0.05). We show that UBF can be considered a value-added product with health-promoting properties. The utilization of UBF as a functional food ingredient will benefit the consumer.

## 1. Introduction

Bananas are one of the most produced and consumed fruits worldwide. They are popular all over the world, especially in tropical regions such as Thailand. Bananas are the fourth largest export product in the world after rice, wheat, and corn. A large number of green bananas (*Cavendish* spp.) are grown in Chiang Rai province in the northern part of Thailand, which are mostly exported to China. The Phaya Mengrai Kankaset Company produced approximately 7300 tons of green bananas per year. However, they also rejected around 1440 tons during the grading process because of greater or lower than acceptable size, as well as over ripening. The cost of rejection under quality standards is more than 2.8 million baht per year (91,650 USD). If this loss after harvesting could be solved in a suitable way, such as the further utilization or added value of these rejected bananas, the company could benefit further and potentially expand their business (personal communication with the owner).

Unripe banana pulp contains up to 70–80% starch [1]. In addition, Sarawong et al. [2] reported that banana flour has a high quantity of resistant starch (approximately 40.9–58.5%) and dietary fiber (6.0–15.5%). Various processed banana products have already been developed, such as sun-dried bananas, banana crisps, or instant green banana flour [3]. Nowadays, many new products made from banana flour with potential commercial value are available. However, a literature search via Scopus over the last decade found only 46 articles about these products. Only two articles applied green banana flour in bread-based products; one article focused on the rheological, textural, and structural changes in dough and bread, while the focused on the effect of three types of flours (including green banana flour) on local dough properties during bread baking. Banana flour can be used in various cakes/bread (wet and dry cakes), as well as for infant feeding. Banana flour does not contain gluten, so it can be used as a functional ingredient in bread making for gluten intolerant consumers [4]. Bread is one of the most popular wheat-based food products; it is made by combining basic ingredients, such as water, wheat flour, yeast, sugar, milk powder, improver, shortening and salt [5]. Flour and water are the most important ingredients in a bread recipe, as they affect the crumb texture. They are primarily responsible for bread structure and bite characteristics [5]. People diagnosed with celiac disease comprise approximately 1% of the world’s population, and adherence to a strictly gluten-free diet is crucial for these patients [6]. So, alternative flours or food ingredients are a key research area and ongoing project for food technologists and nutritionists.

Huang and Bohrer [7] reported that there were positive structural and technological attributes as a result of including banana flour in beef emulsions. Bread incorporated with 10% banana pseudo-stem flour resulted in a lower volume, darker crumb, and lighter crust color [8]. In addition, partially substituting banana pseudo-stem flour for wheat flour in bakery products has the potential to increase dietary fiber intake. There are many reports that mention the beneficial effects of using banana flour on human health. Banana flour is made from rejected unripe bananas after harvest, which are converted into a useful product that can have a positive effect on the economy. Therefore, the aim of this study was to characterize the basic properties of banana flour and starch from unripe bananas. The incorporation of the obtained banana flour in bread at different percentages of substitution (0–80%) was also performed, and quality attributes were monitored.

## 2. Results and Discussion

### 2.1. Chemical Composition of Banana Flour

The chemical compositions of wheat flour (WF) and unripe banana flour (UBF) are shown in Table 1. A high amount of carbohydrates was found in both UBF (86%) and WF (73%). Total dietary fiber, amylose content, ash, and tannins were higher in UBF than in WF. The second-largest component of WF is protein (15%), followed by amylose and moisture content, respectively. A higher fat content was also observed in WF than in UBF, while only UBF contained tannins. The composition of WF and UBF was in accordance with the values reported by Gomes et al. (2016) and Ng et al. (2014). In addition, high dietary fiber in banana flour demonstrates its potential to be applied as a high fiber source in bakery products [9].

The minerals present in WF and UBF include sodium and potassium (Table 1). UBF had a significantly higher sodium content (63.48 mg/100 g wb) and potassium content (1133.90 mg/100 g wb) compared to WF (*p* < 0.05). The content of potassium was higher compared to sodium, and the Na/K ratio was low in UBF. This is considered to be an advantage from a nutritional point of view, since the high intake of sodium chloride and diets with a high Na/K ratio relate to the incidence of hypertension and stroke [10]. Potassium is a mineral that is important for controlling fluid balance in the body. Potassium, along with other minerals such as sodium, calcium, and magnesium, helps to regulate water balance in cells, as well as a blood pressure. Taiwo and Kehinde [11] reported that cardava banana flour samples had a potassium content ranging from 44.76 to 52.88 mg/100 g, while the potassium content of plantain flour samples ranged from 40.45 to 49.53 mg/100 g.

### 2.2. Antioxidant Activity

The 2,2-diphenyl-1-picryl-hydrazyl-hydrate) (DPPH) radical scavenging capacity of the samples is shown in Table 1. UBF showed good antioxidant activity. Bananas have a high total phenolic content, which may explain the strong antioxidant activity seen in UBF [12]. The phenolic content in banana flour was confirmed by Taiwo and Kehinde [11], who reported that the total phenolic content of cardava banana flour samples ranged from 15 to 46 µg GAE/100 g while plantain flour samples had values in the range of 10 to 34 µg GAE/100 g. The DPPH scavenging activity of UBF (38.31 µmole Trolox/100 g db) was higher than that of WF (34.91 µmole Trolox/100 g db) (*p* < 0.05). This result may be related to the content of phenolic compounds in banana flour. The polyphenols reported to be present in banana include gallic acid, catechins and epicatechins, anthocyanins and other flavonoid derivatives [13].

### 2.3. Functional, Physical and Thermal Properties Determination

Functional properties

The solubility, swelling power, and water absorption capacity of WF and UBF are shown in Table 1. There was no significant difference in swelling power and water absorption capacity (*p* > 0.05), while the solubility of WF (3.11%) was significantly higher than that of UBF (2.33%) (*p* < 0.05). The high water content of WF may explain its high solubility in this experiment. The swelling power of the sample may correlate with amylose and amylopectin content. The higher amylose content in UBF (18.56 g/100 g) compared to WF (14.65 g/100 g) results from a crystalline network structure with strong linkage, which restricts swelling. Sasaki and Matsuki [14] concluded that the swelling power of wheat starch correlated negatively with amylose content. They also reported that the amylopectin structure showed that starch with a higher swelling power tends to contain higher proportions of longer chains (degree of polymerization ≥ 35). Starches with varying amylose and amylopectin content are of particular interest due to their ability to influence and modify the texture, quality, and stability of starch-based food products [15]. Aziah et al. [16] reported that dietary fiber from banana flour is able to bind or entrap more water than WF. The high water holding capacity of fiber-rich flour is attributed to the higher number of hydroxyl groups found in the fiber structure, which tends to allow more water interactions through hydrogen bonding. However, the results from this investigation were not in accordance with previous work in the literature. This may be the result of differences in the starting material composition, as well as the process used for preparing the flour.

Color profile

The color attributes of UBF and WF are presented in Table 1 in the form of L*, a*, and b* values and their appearance. The color of UBF (L*, a*, and b* value) varied significantly compared to WF (*p* < 0.05), particularly for the L* value (74.69 for UBF and 93.50 for WF). UBF had higher redness (+a*) and yellowness (+b*) compared to WF. Similar observations were recently described by Kongolo et al. [17]. The color attributes of UBF limit its application.

Starch granules

**Figure 1:**
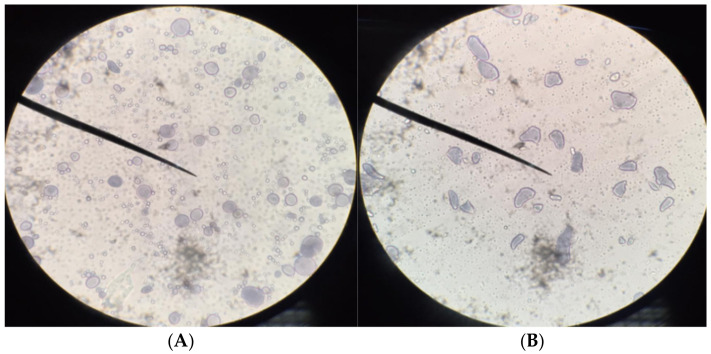
Starch granules (40× magnification) of wheat flour (**A**) and unripe banana flour (**B**).

The microstructure of the starch granules in WF and UBF was analyzed by light microscopy and presented in Figure 1. The starch granules showed irregularly shaped, elongated forms, appearing as ovals with ridges. In addition, the size of the UBF starch granules was much larger than the WF starch granules. Elongated granules of banana starch were 2.75–3.25 µm in width and 4.25–6.25 µm in length, whereas WF granules were 2.5–3.0 µm in length. As reported by Jiang et al. [18], small granules are digested more rapidly than large granules, presumably because of their greater surface area. In addition, the size and shape of starch granules are important factors in determining the potential use of starch; for example, small granules (2–10 µm) can be used as fat substitutes, and large granules can be applied in biodegradable plastic films [19].

Pasting properties

The pasting profiles of UBF and WF were compared to understand their potential industrial applications. The pasting properties varied significantly between the two types of flours (Table 2). The pasting temperature of WF and UBF was 69.82 °C and 82.10 °C, respectively. The peak viscosity of UBF (173.56 Rapid-Visco Analyser units (RVU)) was significantly higher than that of WF (212.58 RVU) (*p* < 0.05). The breakdown viscosity of UBF (173.56 RVU) was significantly higher than that of WF (89.78 RVU) (*p* < 0.05). This results from the flour composition, such as protein, lipid and amylose content. The setback viscosity of WF was significantly higher than that of UBF (*p* < 0.05). The differences in the pasting properties might be associated with the type of drying, the microstructure of the starch particles, and the variation in the starch quantities, including damaged and resistant starch content. These results confirm the expected characteristic of WF to have a higher tendency for retrogradation compared to UBF. As a result, cooked WF possesses the potential to form a gel, whilst cooked UBF can only form a paste upon cooling [20]. The difference in setback might be due to the amount and the molecular weight of amylose leached from the granules and the presence of gelatinized starch remnants [21]. Moreover, the differences in the size and shape of the starch granules could also affect starch properties. UBF exhibited resistance to retrogradation as indicated by the low setback value compare to WF.

Thermal properties

The gelatinization temperature (Tp) and enthalpy of gelatinization (ΔH) for WF and UBF using differential scanning calorimetry (DSC) are given in Table 2. The Tp of UBF (77 °C) was significantly higher than WF (65 °C) (*p* < 0.05). The differences in gelatinization temperature might be attributed to the differences in amylose content and the size, form, and distribution of starch granules. The internal arrangement of starch fractions within the granules could also play a role [18]. The ΔH for UBF and WF is 5.00 and 0.69 (J/g), respectively. This confirmed the observations of Jiang et al. [18], who reported that more crystalline component quantities would lead to higher gelatinization enthalpies.

### 2.4. Bread Quality

Loaf volume and density of bread

The quality attributes of bread, including appearance, loaf volume, and density, are indicated in Table 3. The substitution of banana flour in the recipe caused a significant decrease in loaf volume (*p* < 0.05). According to a report by Feili et al. [9], this could be explained by the fact that substitution with banana flour can cause gluten dilution, which consequently affects optimal gluten matrix formation during the mixing, fermentation, and baking steps. Moreover, the addition of UBF caused a significant increase in the density of bread (*p* < 0.05). Hosokawa et al. [22] demonstrated that banana flour prepared from banana fruit stored at 20 °C for 2 weeks could successfully be used as a substitute for gluten or hydrocolloids, indicating its utility as an ingredient for gluten-free bread making. They also concluded that ripe banana flour was shown to have good potential as a substitute for gluten. However, according to our results, the addition of a high level of UBF affects the color properties of the final product, which may not be acceptable to the consumer. Moreover, the bread made in this study cannot be sold as gluten-free bread because the WF in the recipe was not totally substituted.

Texture profile analysis

The texture profiles of breads with and without UBF are shown in Table 4. The hardness of the bread was significantly increased with increasing UBF content from 245 g (100% WF) to 615 g when UBF was added up to 80% (*p* < 0.05). The incorporation of UBF resulted in a more compact bread with a denser structure. Adhesiveness was not affected by the UBF. In contrary, cohesiveness and adhesiveness were significantly affected by the addition of UBF (*p* < 0.05), indicated by the increase in value from 0.55 to 0.27 g.s and −0.54 to −1.35 g.s when WF was replace by UBF up to 80%, respectively. This reduction indicated that bread formulated with UBF has low resistance before the bread structure is deformed by teeth upon consumption. The springiness of the bread was significantly reduced by the addition of UBF (*p* < 0.05). The springiness of bread containing 10%, 20% and 40% UBF was not significantly different (*p* < 0.05). According to a report by Feili et al. [9], the interaction between gelatinized starch and gluten dough, which cause the dough to be more elastic, can form a continuous sponge structure in bread after heating. A lower amount of gluten results in a lower ability to hold gases, which caused a reduction in elasticity in the bread. Bread samples substituted with UBF showed significantly higher values for gumminess and chewiness (*p* < 0.05). Feili et al. [9] showed a similar trend for breads made with additional fiber.

## 3. Materials and Methods

### 3.1. Materials

Unripe green bananas (*Cavendish* spp.) were obtained from Phaya Mengrai Kankaset Company (Chiang Rai, Thailand). Wheat flour (White Swan brand) and other basic ingredients (such as water, yeast, sugar, milk powder, improver, shortening, and salt) were purchased from Makro hypermarket (Chiang Rai, Thailand). 2,2-Dipheny1-1-picryl hydrazyl (DPPH) was purchased from Sigma-Aldrich (St. Louis, MO, USA). Sodium chloride, acetic acid, sodium hydroxide, ethanol, and 6-hydroxy-2,5,7,8-tetramethylchroman-2-carboxylic acid (Trolox) were purchased from Calibiochem (Darmstadt, Germany).

### 3.2. Methods

#### 3.2.1. Banana Flour Preparation

Banana flour was prepared according to the method of Alves et al. [23] with some modifications. Green banana fruits were dipped into 50 ppm chlorine, washed, peeled, cut into 5 mm slices, and immediately dipped in a 0.1% (*w*/*v*) citric acid solution for 15 min. Banana fruit slices were dried at 40 °C for 16 h in a tray dryer. The dried samples were ground in a pulverizing machine for 90 s and passed through 60 mesh sieves. The flour obtained was stored at −20 °C in a vacuum sealed plastic bag before further testing.

#### 3.2.2. Proximate Composition and Chemical Properties Analysis

Moisture content was analyzed according to the official method outlined in Association of Official Agricultural Chemists (AOAC) 930.15. Ash, protein, and fat were analyzed according to the official method outlined in AOAC 942.05, 954.01, and 920.39, respectively [24]. Carbohydrates were calculated by subtracting moisture, fat, protein, and ash contents from 100. All samples were analyzed in triplicate.

Total dietary fiber

Dietary fiber content was determined by an enzymatic–gravimetric method according to AOAC method 985.29 [24]. The dried and fat-free samples were gelatinized with heat stable *α*-amylase and then enzymatically digested with protease and amyloglucosidase to remove the protein and starch present in the sample. Ethanol was added to precipitate the soluble dietary fiber. The residue was then filtered and washed with ethanol and acetone, then dried and weighed. Total dietary fiber was calculated as the weight of the residue minus the weight of the protein and ash and reported as a percentage of the original sample weight.

Amylose content

Amylose content was determined using the method of Hassan et al. [25]. Briefly, 0.10 g of sample was weighed into a 100 mL volumetric flask. Then, 1 mL of 95% ethanol and 9 mL of 1 M sodium hydroxide solution were added. The contents were thoroughly mixed, and the sample solution was heated for 10 min in boiling water. After cooling, the solution was topped up with distilled water and thoroughly shaken. The starch solutions were then treated with 1.0 mL of 1 M acetic acid and 2.0 mL of iodine solution. The solution was diluted with distilled water and the absorbance was read using a UV-Vis spectrophotometer (G105 UV-VIS, Thermo Scientific Inc., Walthamm, MA, USA) at 620 nm. The absorbance of blank solution, prepared accordingly, was subtracted from that of the sample and amylose contents were calculated.

Tannin content

Tannin content analysis was performed according to the method of Atanassova et al. [26]. Briefly, 3 g of sample was extracted with distilled water into a 250 mL volumetric flask for 4 h at room temperature, and then samples were filtered. Twenty-five mL of the infusion was measured into a 1 L conical flask, and then 25 mL of indigo solution and 750 mL distilled water was added. An aqueous solution of KMnO_4_ (0.1 N) was used for titration until the blue colored solution changed to green. Then, a few drops at a time were added until the solution turned golden/yellow. A standard solution of indigo carmine was prepared as following: 6 g indigo carmine was dissolved in 500 mL of distilled water by heating; after cooling, 50 mL of 95–97% H_2_SO_4_ was added, and the solution was diluted to 1 L and filtered. The blank was tested by the titration of a mixture of 25 mL indigo carmine solution and 750 mL distilled water. All samples were analyzed in triplicate.

Sodium and potassium content

Sodium and potassium content were determined using a modified method reported by Chen et al. [10]. Briefly, 2 g of sample was placed in a porcelain crucible and ashed in an oven at 650 °C for 4 h. The ashed material was dissolved in 10 mL HCl solution (1:1, HCl:H_2_O) and diluted with distilled water up to 25 mL. Sodium and potassium were analyzed by an atomic absorption spectrometer.

Antioxidant activity

Antioxidant activity was determined using a modified method reported by Savlak et al. [27]. Briefly, 1 g of samples was mixed with 25 mL methanol:water mixture (*v/v*, 1:1) and incubated at 50 °C for 15 min in an oscillating water bath (Memmert, D-91126, Schwabach, Germany). The mixture was centrifuged at 4000 rpm (LegendX1R, Thermo Fisher Scientific, Germany) at 20 °C for 10 min. The supernatant was transferred into a 100 mL volumetric flask. The same procedure was then repeated twice. The volume of the flask was topped up to 100 mL with methanol:water mixture (*v/v*, 1:1). The content of the volumetric flask was filtered through Whatman No. 4 filter paper (GE Healthcare, Maidstone, UK). The samples (50 µL) were mixed with a 2 mL aliquot of 60 µM DPPH radicals in methanol. Distilled water was used as a control instead of extract. The reaction mixture was vortexed and left to stand at 25 °C in the dark for 60 min. Absorbance at 517 nm was measured using a UV-Vis spectrophotometer (G105 UV-VIS, Thermo Scientific Inc., MA, USA) with methanol as the blank. The control and standard were subjected to the same procedures as the sample except that, for the control, only distilled water was added, and for the standard, the extract was replaced with 0–1000 µM Trolox standard.

#### 3.2.3. Determination of Functional, Physical and Thermal Properties

The solubility, swelling power, and water absorption capacity of the samples were determined using a modified method from Torre-Gutie’rrez et al. [28]. Briefly, 40 mL of a 1% sample solution (*w/v*) was prepared in a 50 mL centrifuge tube. A magnetic agitator was put in the tube, which was placed in a water bath for 30 min at 90 °C. The suspension was then centrifuged at 5000× *g* for 10 min (LegendX1R, Thermo Fisher Scientific, Germany), then the supernatant was decanted and the swollen granules were weighed. A 10 mL sample was taken from the supernatant, placed in a crucible, and dried in an air convection oven at 120 °C for 4 h (Memmert, D-91126, Schwabach, Germany). Solubility, swelling power, and water absorption capacity were then calculated.

Color determination

Color attributes of banana flour and wheat flour were measured with a Hunterlab LabScan (Hunter Lab, VA, USA) and reported using the CIELAB color scale. The CIELAB color scale with the parameters L*, a*, and b* indicates lightness on a scale of 0–100, with 0 representing black and 100 representing white. Coordinate a* corresponds to red (+) and green (−) while b* corresponds to yellow (+) and blue (−) [12].

Starch granules determination

Starch granules were observed using the method of Eduardo et al. [29]. The water slurries of banana flour and wheat flour (soaked overnight at room temperature) were smeared onto a glass slide. After drying, the samples were stained with iodine solution and covered with a glass coverslip. The samples were thereafter examined with a light microscope using a 40× magnification (DMLS, Leica, Solms, Germany).

Pasting properties determination

Pasting properties of the samples were determined according to the method of Mesquita et al. [30] with a rapid viscoanalyzer (RVA 4500, Chapa Techcenter, Bangkok, Thailand) using 10% starch suspensions (*w/w*) with a total mass of 27.5 g. The program ‘starch 1’ was used (50 °C for 1 min, heating from 50 to 95 °C at a rate of 6 °C min^−1^, maintaining the paste at 95 °C for 5 min, cooling from 95 to 50 °C at a rate of 6 °C min^−1^). The viscosity was expressed as RVU (1 RVU = 12 cp). From the graph obtained, pasting temperature, maximum viscosity (peak), breakdown, final viscosity, and setback were evaluated. This analysis was carried out in triplicate.

Thermal properties determination

Thermal properties were determined according to the method of Jiang et al. [18] by using differential scanning calorimetry (DSC 822e, Mettler Toledo, Bangkok, Thailand). Samples (2 mg) were weighed into a DSC aluminum pan and then which was added by distilled water (4 mL). The DSC pans were hermetically sealed and equilibrated for 12 h. Starch dispersions were gelatinized from 20 to 100 °C at a heating rate of 10 °C/min. An empty pan was used as a reference. The gelatinization or peak temperature (Tp) and the transition enthalpy (ΔH) were obtained directly using the accompanying Pyris software. Triplicate measurements were carried out.

#### 3.2.4. Bread Preparation

The different bread formations are shown in Table 5 and prepared according to the method of Phogthia et al. [31] with slight modifications. Flour, milk, and instant yeast were sifted into bowl, while salt and sugar were dissolved in milk. The sifted dry ingredients were mixed with egg and the milk mixture until all of the milk absorbed into the dry mixture. Butter was added to the mixture and the dough kneaded until the mixture become soft and smooth in 6 min at the speed of 2 in a mixer (RN10/VL2 planetary mixer, A/S Wodschow & Co., Broendby, Denmark). The mixture was left to undergo fermentation for 1 to 1.5 h at 30 °C, 85% RH for 30 min in a fermentation chamber (G66W, MANZ Backtechnik GmbH, Creglingen, Germany). The dough was divided into the desired weights, then rolled to obtain a smooth surface. The dough was put into a loaf shape pan and allowed to develop for about 40–60 min, or until the dough had risen up to fill the pan. The dough was baked at 200 °C for about 35 min, or until it was completely cooked.

#### 3.2.5. Determination of Bread Quality

Loaf volume and density

Loaf volume was measured 50 min after loaves were taken out of the oven using the rapeseed displacement method reported by Feili et al. [9]. Briefly, rapeseeds were poured into a container to measure the volume, and then were measured in a graduated cylinder and marked. Thereafter, a sample was placed in the same container and seeds were added until the bread was covered. Again, the rapeseeds were measured in a graduated cylinder and marked. The recorded values were calculated and reported as the loaf volume of bread. The bread density was also calculated.

Texture profile analysis

The texture parameters (hardness, adhesiveness, springiness, cohesiveness, gumminess, and chewiness) of the bread samples were measured objectively using a texture analyzer (TA-XT-plus, Chapa Techcenter, Bangkok, Thailand) according to the method of Feili et al. [9]. All samples were prepared and baked on the day of the test. The probe was calibrated according to the instructions before conducting the test. A cube sample (2 cm × 2 cm × 2 cm) was cut from the middle of the bread sample and placed centrally beneath the probe *(p*/36 cylinder probe; 36 mm). The compression test was selected and performed using a 5 kg load cell; the sample was compressed to 45% of its original height. The strain required for 45% compression was recorded using the following conditions: pretest speed = 1.0 mm/s; test speed = 1.7 mm/s; post-test speed = 10 m/s; compression distance = 25%; and trigger type = auto (5 g). The values reported are the average of three readings.

#### 3.2.6. Statistical Analysis

Data were expressed as means ± standard deviation. The one-way analysis of variance (ANOVA) by Duncan’s test (*p* < 0.05) was calculated using the SPSS 18.0 statistical software program (SPSS 16.0 for Windows, SPSS Inc., Chicago, IL, USA).

## 4. Conclusions

The banana flour from unripe *Cavendish* spp. can be used as a functional ingredient for bread making. The physico-chemical properties are comparable with wheat flour but also showed a higher content of some components, particularly minerals, fiber and bioactives. Banana flour can be used to substitute wheat flour to a degree. According to the concept of sustainability and feasibility, this work contributes to the reduction of food loss and food waste by turning a rejected food source into a highly commercial output in order to sustain this renewable resource.

## Figures and Tables

**Table 1:** Chemical composition, physico-chemical properties, and functional properties of wheat flour and unripe banana flour.

Composition/ Properties	Wheat Flour	Unripe Banana Flour
Appearance	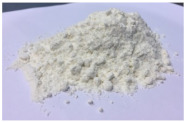	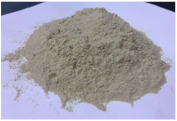
Moisture content ^A^	10.55 ± 0.04 ^a^	6.11 ± 0.17 ^b^
Ash ^A^	0.49 ± 0.01 ^b^	1.83 ± 1.58 ^b^
Protein ^A^	14.82 ± 0.34 ^a^	4.98 ± 0.73 ^b^
Fat ^A^	0.85 ± 0.14 ^a^	0.34 ± 0.05 ^b^
Carbohydrates ^A^	73.29 ± 0.34 ^b^	85.81 ± 0.58 ^a^
Total dietary fiber ^A^	2.79 ± 0.79 ^b^	7.11 ± 0.85 ^a^
Amylose ^A^	14.65 ± 0.87 ^b^	18.56 ± 0.56 ^a^
Tannin ^A^	N.D.	0.14 ± 0.00
Sodium ^B^	55.65 ± 0.35 ^b^	63.48 ± 2.6 ^a^
Potassium ^B^	118.05 ± 0.71 ^b^	1133.90 ± 3.46 ^a^
Radical scavenging activity ^C^	34.91 ± 0.25 ^b^	38.31 ± 0.37 ^a^
L*	93.50 ± 0.16 ^a^	74.69 ± 1.00 ^b^
a*	0.77 ± 0.03 ^b^	2.60 ± 0.05 ^a^
b*	7.59 ± 0.14 ^b^	10.39 ± 0.20 ^a^
Solubility (%, *w/w* db)	3.11 ± 0.01 ^a^	2.33 ± 0.30 ^b^
Swelling power (%, *w/w* db) ^ns^	3.93 ± 0.64	3.37 ± 0.67
Water absorption capacity (%, *w/w* db) ^ns^	0.55 ± 0.07	0.46 ± 0.39

Values are expressed as means ± SD (*n* = 3); different superscript letters (a, b) in the same row indicate significant difference at *p* < 0.05; ns = not significant; N.D. = not detected; ^A^ g/100 g wb; ^B^ mg/100 g; ^C^ μmole Trolox/100 g db; ns = not significant. L*, a*, b*: CIELab color system.

**Table 2:** Thermal and pasting properties of wheat flour and unripe banana flour.

Properties	Wheat Flour	Unripe Banana Flour
Pasting temperature (°C)	69.82 ± 0.45 ^b^	82.10 ± 0.48 ^a^
Peak viscosity (RVU)	212.58 ± 0.30 ^b^	493.53 ± 0.21 ^a^
Breakdown viscosity (RVU)	89.78 ± 1.43 ^b^	173.56 ± 2.59 ^a^
Final viscosity (RVU)	235.39 ± 1.35 ^b^	421.89 ± 3.56 ^a^
Setback viscosity (RVU)	112.61 ± 0.27 ^a^	98.03 ± 2.87 ^b^
Gelatinization temperature (Tp)	65.53 ± 1.93 ^b^	77.22 ± 0.81 ^a^
Gelatinization enthalpy (∆H)	0.69 ± 0.21 ^b^	5.00 ± 0.06 ^a^

Values are expressed as means ± SD (*n* = 3); different letters in the same row indicate significant difference at *p* < 0.05; RVU = rapid viscoanalyzer units.

**Table 3:** Appearance, loaf volume, and density of breads with different amounts of unripe banana flour.

Banana Flour (%)	Appearance		Loaf Volume (mL)	Density (g/mL)
0 *	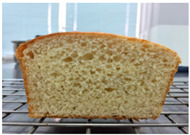	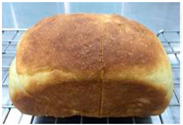	249.00 ± 3.61 ^a^	1.91 ± 0.03 ^f^
10	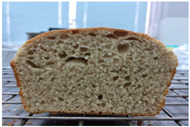	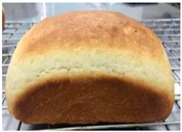	220.33 ± 2.51 ^b^	2.12 ± 0.02 ^e^
20	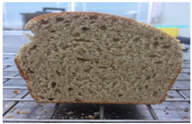	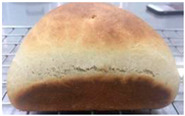	179.67 ± 3.51 ^c^	2.53 ± 0.05 ^d^
40	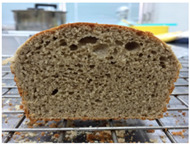	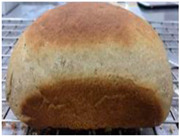	160.00 ± 2.00 ^d^	2.72 ± 0.03 ^c^
60	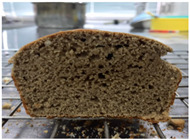	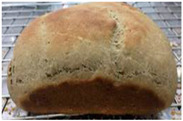	142.67 ± 2.52 ^e^	3.03 ± 0.06 ^b^
80	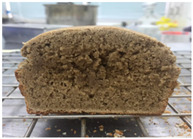	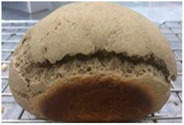	102.33 ± 2.52 ^f^	4.46 ± 0.11 ^a^

* WF 100%; values are expressed as means ± SD (*n* = 3); different letters in the same column indicate significant difference at *p* < 0.05.

**Table 4:** Texture profile analysis of breads made with different amounts of unripe banana flour.

Banana Flour (%)	Hardness (g)	Adhesiveness ^ns^ (g.s)	Springiness	Cohesiveness	Gumminess	Chewiness
0 *	245.33 ± 1.57 ^f^	−0.54 ± 0.27	0.89 ± 0.01 ^a^	0.55 ± 0.02 ^a^	115.15 ± 1.10 ^e^	104.31 ± 1.75 ^d^
10	276.09 ± 1.58 ^e^	−0.53 ± 0.06	0.75 ± 0.17 ^a, b, c^	0.55 ± 0.06 ^a^	146.73 ± 0.66 ^c^	114.42 ± 1.48 ^b, c^
20	296.60 ± 0.91 ^d^	−1.11 ± 0.37	0.82 ± 0.01 ^a, b^	0.47 ± 0.02 ^b^	144.89 ± 1.07 ^d^	115.85 ± 1.58 ^b^
40	324.19 ± 1.34 ^c^	−1.60 ± 1.62	0.70 ± 0.05 ^a, b, c^	0.35 ± 0.02 ^c^	170.05 ± 1.56 ^b^	126.22 ± 1.72 ^a^
60	463.11 ± 1.90 ^b^	−1.30 ± 1.13	0.57 ± 0.12 ^c^	0.30 ± 0.07 ^c, d^	106.00 ± 1.78 ^f^	54.81 ± 1.77 ^e^
80	615.09 ± 1.54 ^a^	−1.35 ± 0.07	0.64 ± 0.08 ^b, c^	0.27 ± 0.03 ^d^	184.01 ± 1.32 ^a^	112.27 ± 1.58 ^c^

* WF 100%; Values are expressed as means ± SD (*n* = 3); Different letters in the same column indicate significant difference at *p* < 0.05; ns = not significant.

**Table 5:** Formulation of bread making with different amounts of wheat and unripe banana flour.

Ingredients	Banana Flour (%)					
	0%	10%	20%	40%	60%	80%
Banana flour	0	50	100	200	300	400
Wheat flour	500	450	400	300	200	100
Milk	280	280	280	280	280	280
Sugar	80	80	80	80	80	80
Salt	5	5	5	5	5	5
Instant yeast	10	10	10	10	10	10
Egg	37.5	37.5	37.5	37.5	37.5	37.5
Butter	100	100	100	100	100	100

## Data Availability

Data sharing is not applicable.

## Sample Availability

Not available.
